# Small primary adenocarcinoma in adenomyosis with nodal metastasis: a case report

**DOI:** 10.1186/1471-2407-7-103

**Published:** 2007-06-20

**Authors:** Giacomo Puppa, Makio Shozu, Tiziana Perin, Kazuhito Nomura, Annunziata Gloghini, Elio Campagnutta, Vincenzo Canzonieri

**Affiliations:** 1 Division of Pathology, Centro di Riferimento Oncologico, Istituto Nazionale Tumori – IRCCS, Via Pedemontana Occidentale 12, 33081 Aviano PN, Italy; 2 Graduate School of Chiba University, Reproductive Medicine, Chuo-ku, Chiba, Japan; 3 Department of Obstetrics and Gynecology, Kanazawa University School of Medicine, Takara-machi, Kanazawa, Japan; 4 Diagnostic Immunohistochemistry and Molecular Pathology Unit, Istituto Nazionale Tumori – IRCCS, Aviano PN, Italy; 5 Division of Gynecologic Oncology, Centro di Riferimento Oncologico, Istituto Nazionale Tumori – IRCCS, Aviano PN, Italy

## Abstract

**Background:**

Malignant transformation of adenomyosis is a very rare event. Only about 30 cases of this occurrence have been documented till now.

**Case presentation:**

The patient was a 57-year-old woman with a slightly enlarged uterus, who underwent total hysterectomy and unilateral adnexectomy. On gross inspection, the uterine wall displayed a single nodule measuring 5 cm and several small gelatinous lesions. Microscopic examination revealed a common leiomyoma and multiple adenomyotic foci. A few of these glands were transformed into a moderately differentiated adenocarcinoma. The endometrium was completely examined and tumor free. The carcinoma was, therefore, considered to be an endometrioid adenocarcinoma arising from adenomyosis. Four months later, an ultrasound scan revealed enlarged pelvic lymph nodes: a cytological diagnosis of metastatic adenocarcinoma was made.

Immunohistochemical studies showed an enhanced positivity of the tumor site together with the neighbouring adenomyotic foci for estrogen receptors, aromatase, p53 and COX-2 expression when compared to the distant adenomyotic glands and the endometrium. We therefore postulate that the neoplastic transformation of adenomyosis implies an early carcinogenic event involving p53 and COX-2; further tumor growth is sustained by an autocrine-paracrine loop, based on a modulation of hormone receptors as well as aromatase and COX-2 local expression.

**Conclusion:**

Adenocarcinoma in adenomyosis may be affected by local hormonal influence and, despite its small size, may metastasize.

## Background

Leiomyoma and adenomyosis are both commonly encountered in hysterectomy surgical specimens, often as incidental findings. A frequent association of adenomyosis with other hormone-dependent uterine lesions, both benign and malignant, such as endometrial hyperplasia, endometrial carcinoma and leiomyoma has already been described in the literature [1,2]. However, the development of cancer from adenomyosis is a relatively rare occurrence. In all cases, the following Sampson's criteria should be fulfilled to confirm the malignant transformation:

1) evidence of pre-existing endometriosis at the site of the supposed malignant lesion;

2) exclusion of the possibility of the carcinoma representing an invasion or metastasis from another location;

3) evidence of transitions between the benign and malignant glandular structures; and

4) both glands and stroma must be present to constitute genuine adenomyosis [3].

At present, only about 30 cases of adenocarcinoma arising from adenomyosis without endometrial malignancy have been reported in the English literature [4-12]. In all these cases, the immunohistochemical studies were of limited extent and the few cases reported with immunohistochemistry were all negative for ER [6,9,12].

We report on the clinico-pathological and immunohistochemical findings of a small endometrioid adenocarcinoma arising from adenomyosis, incidentally discovered around a leiomyoma after extensive sampling. To the best of our knowledge, this is the single smallest reported instance of such an occurrence with a distinct immunohistochemical profile. The possible connection between malignant transformation of adenomyosis and benign, hormone-dependent disease is discussed.

## Case presentation

### Clinical summary

The patient was a 57-year-old nulliparous, asymptomatic woman of normal weight. She was in follow-up for a fibromatous uterus. On palpation, a uterine softening was noted. An ultrasound scan displayed the enlargement of a pre-existent leiomyomatous nodule and multiple minute focal lesions throughout the uterine wall, diagnosed as a likely adenomyosis. Menopause had commenced at the age of 54. Her past medical history was significant for papillary serous cystoadenoma of the left ovary which was removed when she was 33 years of age. For the last few years, the patient was being treated with an antihypertensive therapy, while, nine years previously, for a period of two years, she had taken symptomatic therapy for perimenopausal symptoms including progesterone, and, during the last year, a non-hormonal therapy with a hyperprolactinemic effect to reduce hot flushes.

Because of the recent appearance of focal lesions in the uterine corpus in an already enlarged uterus (as seen on the ultrasound scan), a total hysterectomy and unilateral adnexectomy were performed. Four months later, an ultrasound scan revealed an enlargement of the left external iliac chain nodes and a thickening of the lateral pelvic wall up to the inguinal ring. A second-look surgical inspection as well as an extensive clinical work-up, which included a thoraco-abdominal CT scan, excluded the presence of other primary tumors.

## Methods

The tissues were fixed in Bouin solution and embedded in paraffin for histologic processing. Tissue sections were stained with hematoxylin and eosin for conventional histology.

Immunohistochemical stainings were performed using a combination of the avidin-biotin complex peroxidase method (ABC kit; Vector Laboratories, Burlingham, CA, USA) and microwave antigen retrieval with the following antibodies: ER (Clone 6F11, Novocastra, Newcastle, UK); PR (Clone Pgr636 Dakocytomation, Glostrup, Denmark); C-erb B2 (Clone A0485, polyclonal antibody Dakocytomation, Glostrup, Denmark); Cyclooxygenase-2 (COX-2) (clone 4H12, Novocastra, Newcastle, UK); p53 (Clone DO-7, Novocastra, Newcastle, UK) and CA125 (Clone Ov185:1, Novocastra, Newcastle, UK).

The sections were also stained for aromatase as follows: sections were dewaxed in xylene and taken through a graded series of ethanol. Epitope retrieval was performed by enzymatic digestion with trypsin for 20 minutes at 37°C (TRYPSIN Tablets; Sigma, St. Louis, MO, USA). After incubation with 0.3% hydrogen and protein block solution (DAKO) for 5 minutes, the sections were treated with polyclonal antiaromatase antibody (provided by Dr. Nobuhiro Harada, Ph.D., Fujita Health University, Nagoya, Japan) for 15 minutes at room temperature following dilution to 1:1,000 in buffer. After incubation with the primary antibody, streptavidin-biotin complex and streptavidin-peroxidase complex were applied for 15 minutes each at room temperature. Colour was developed by incubation with 3,3-diaminobenzidine-tetrahydrochloride for 1 min, followed by counterstaining with hematoxylin.

Negative controls included sections incubated with normal rabbit serum instead of the primary antibody. In addition, an immunoabsorption test was done as follows: a complex of 1 μl of anti-aromatase antibody and 1.5 μl of microsome (at a concentration of 4 μg/μl), extracted from placental tissue, was stirred in phosphate buffer saline containing 1% bovine serum albumin for 12 hours at 4°C, and the resulting precipitate was then centrifuged (10,000 g for 1 hour). Instead of the primary antibody, the supernatant was used for immunostaining.

### Pathological findings

**Figure 1:**
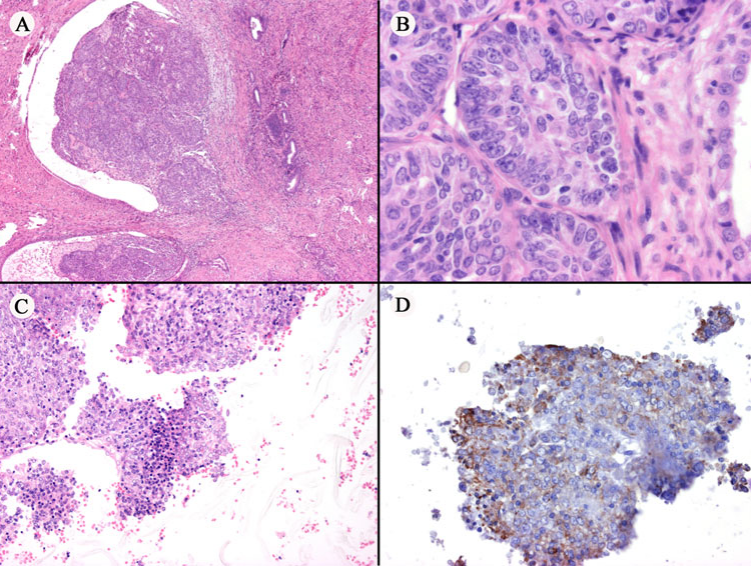
**(A) **The tumor arises from the confluence of transformed foci. **(B) **Higher power magnification of the tumor. **(C) **Cell block cytology of the lymph node metastasis. (**D**) Immunocytochemical staining of the metastasis for CA125.

On gross examination, the uterus measured 7 × 4 × 9 cm and the right ovary measured 3 cm. On cut section, the myometrium of the fundus presented a subserosal whorled leiomyomatous nodule which measured 5 cm, was well circumscribed, solid and surrounded by gelatinous foci. The endometrium appeared thinned with no superficial irregularities. It was totally examined, and only atrophic-cystic changes with focal proliferative activity were seen. There were several adenomyotic foci in the inner half of the myometrium, while some others, in the outer half, were pushed out by the leiomyoma and were located proximally to the serosa and parametrium. A cluster of these glands harboured a focal malignant transformation of the epithelial component, diagnosed as moderately differentiated endometrioid adenocarcinoma measuring 0,8 cm (Figures 1A and 1B) associated with atypical hyperplasia. The tumor was considered invasive because of the presence of solid areas with the confluence of neoplastic glands uninterrupted by stroma.

The enlarged iliac lymph nodes, detected 4 months after surgery, were biopsied by fine-needle aspiration during a second-look procedure, and were diagnosed as metastatic adenocarcinoma (Figure 1C).

**Figure 2:**
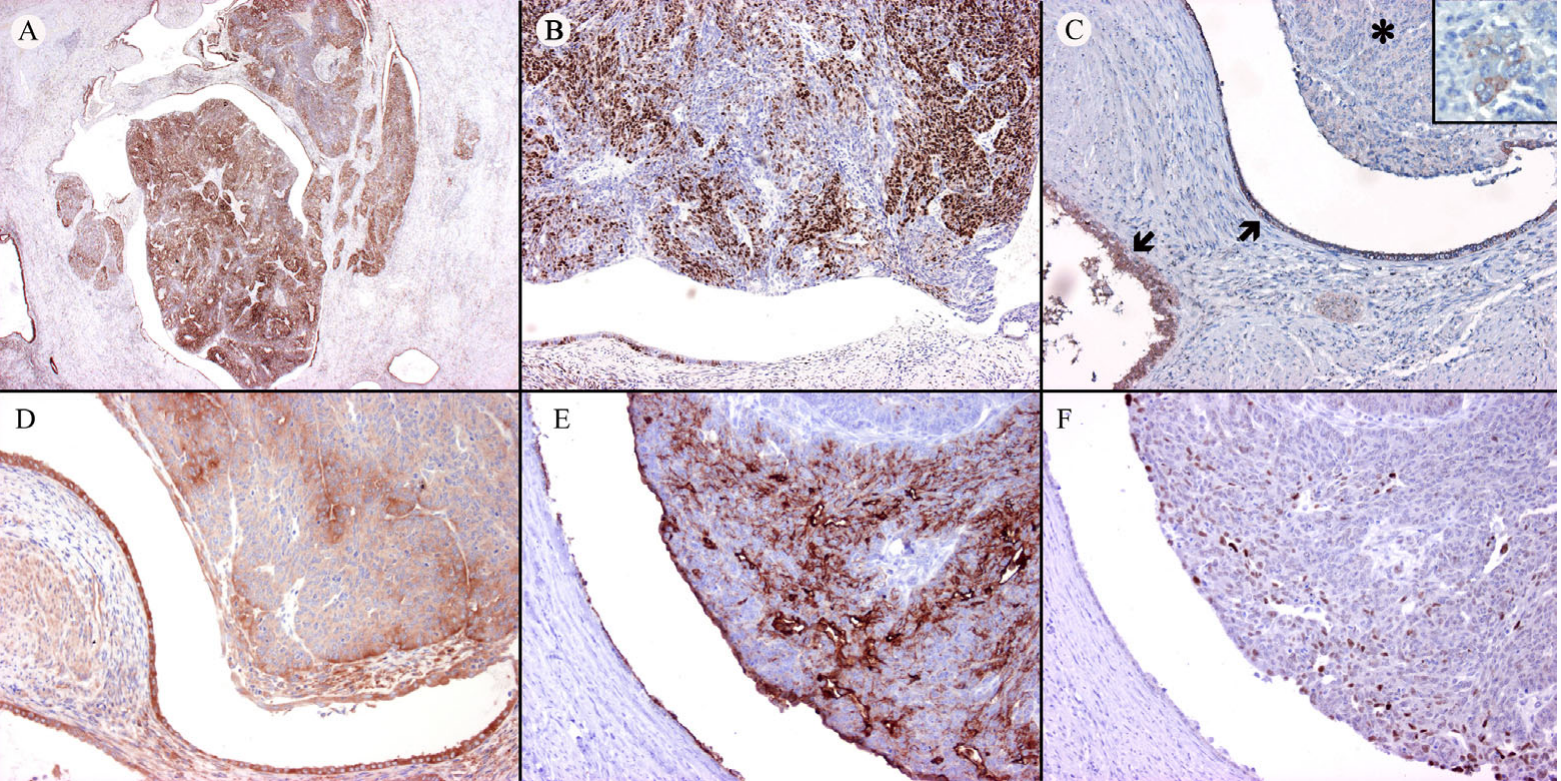
**(A) **Diffuse strong positivity for ER of the tumor. **(B) **Patchy positivity for PR of the tumor. **(C) **Aromatase positivity of benign adenomyotic foci (arrows). Most neoplastic cells are negative for aromatase (asterisk) except for some glands (insert). **(D) **COX-2 positivity of the tumor and adenomyosis. (**E**) CA125 positivity of the tumor and of the adenomyotic epithelium. (**F**) Focal positivity for p53 of the tumor.

**Figure 3:**
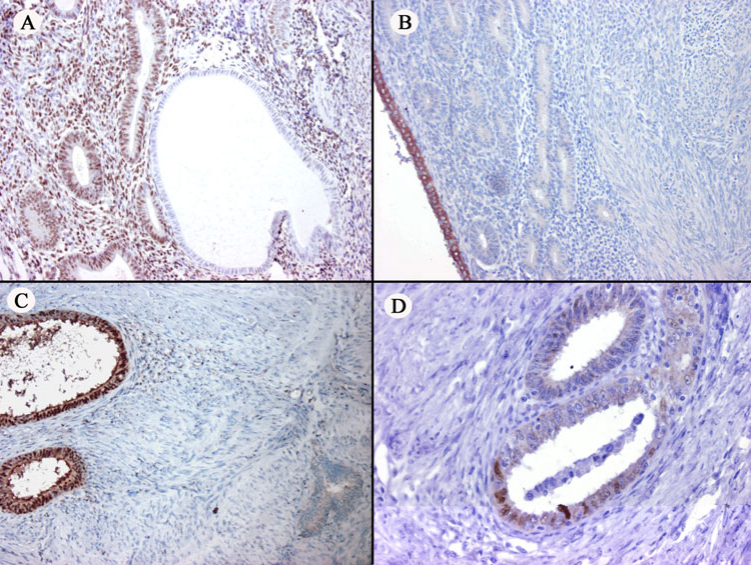
**(A) **The atrophic and proliferative component in the endometrium, respectively negative and positive for ER. **(B) **Aromatase positivity in the superficial layer of a part of the endometrium. **(C) **The adenomyotic glands throughout the uterus: aromatase expressed by some glands (left), whereas other glands are negative (right) **(D) **p53 positivity in some adenomyotic glands.

Immunohistochemically, the primary tumor displayed strong positivity for ER, PR, COX-2, CA125 and focal positivity for p53 and aromatase (Figures 2A–F). The leiomyoma was positive for ER, PR, weakly positive for COX-2 and negative for p53 and CA125. The primary tumor was negative for C-erb B2. In both the eutopic and ectopic endometrial glands, the positivity for PR was diffuse, whereas an alternation of positive and negative foci were noted for ER, aromatase and p53 (Figures 3A–D). In particular, we noted a tendency of the endometrial glands to be negative for ER in the atrophic areas and positive in the proliferative component (Figure 3A). The cell block cytology of the lymph node metastasis was positive for Cytokeratins AE1/AE3, ER, PR, CA125, p53 and COX-2 (Figure 1D, only CA125 shown).

## Conclusion

The case that we present is a single, small, moderately differentiated endometrioid adenocarcinoma which developed into adenomyosis with subsequent metastatic spread to the iliac lymph nodes. The primary tumor was positive for ER, PR, COX-2, CA125, p53 and focally weak-positive for aromatase. Malignant transformation of adenomyotic foci is *per se *a rare event, but the development of metastatic lymph nodal deposits from a very small primary adenomyotic adenocarcinoma is quite exceptional.

The diagnosis of malignant tumors in adenomyosis is usually made in an advanced stage of disease when the neoplasm has grown in nodules within the uterine wall or has involved the endometrium causing abnormal uterine bleeding and/or has spread out of the uterus [5,7-9,12].

In 1925, Sampson introduced the histological criteria to define an adenocarcinoma arising in external endometriosis, which can be adapted to adenomyosis [3].

In the literature, the clinico-pathological findings of 8 cases of adenocarcinoma arising in adenomyosis were presented by Hernandez and Woodruff in 1980, but these were all associated with endometrial adenocarcinomas. They also evaluated 16 cases previously reported by other authors in the literature, and found that only 8 of these were not associated with endometrial adenocarcinomas [5]. In the few cases described as small or microscopic tumors, the pathology report did not include the number or the diameter of the largest tumor, mentioning only that the inner or the outer part of the uterine wall was involved [5,11].

It is well known that gynaecological malignancies, including breast, ovarian and uterine carcinomas, are influenced by hormonal stimulation. In the uterus, a peculiar hormonal microenvironment may explain different pathologic conditions, both benign and malignant. The complex inter-relationships between adenomyosis, endometriosis, leiomyomata, endometrial hyperplasia and carcinoma arising in the endometrium or in adenomyosis have been illustrated together with their hormonal dependency [1,2,13], and factors like obesity and hormonal replacement therapy have been considered as relevant risk factors [13,14].

In postmenopausal women, estrogen formation is ensured by extraglandular tissues, namely adipose tissue and skin. Endometriosis and leiomyomas constitute two added sources of aberrant estrogen biosynthesis, primarily by aromatase enzymatic activity, promoting their self-stimulation [15,16]. Aromatase detection has already been proposed as a test for endometriosis in endometrial biopsies because aromatase cytochrome P450 is expressed in the eutopic endometrium of patients with endometriosis but not in those of disease-free women [17].

In endometriosis, aromatase activity is stimulated by prostaglandin E(2) wich, in turn, is up-regulated by increased levels of the enzyme cyclo-oxygenase-2 (COX-2) [18].

Finally, besides aberrant aromatase expression, a local estrogen overproduction in endometriosis may also be a result of a deficiency of 17β-hydroxysteroid dehydrogenase, resulting in failure to metabolize 17β-Estradiol [19].

In three studies, adenocarcinomas in adenomyosis were reported as all negative for ER and fairly negative for PR [6,9,12]. The reported cases in these small series were rather advanced. Therefore, the hormone receptor and C-erb B2 status in our case of moderately differentiated adenocarcinoma is indicative of early tumor development, similar to that observed in correspondent neoplasms arisen in the eutopic endometrium where hormonal receptor positivity and C-erb B2 negativity are most often associated with low grade and early-stage tumors [20,21]. A peculiar observation in our case is the alternation of positive and negative areas for ER, aromatase and COX-2 in both the normal and the pathological tissues, including the endometrium and the adenomyotic foci. This heterogenous, "checker-board-like" expression of steroid receptors and enzymes involved in steroidogenesis suggests firstly that there are different fields of hormonal responsiveness, and secondly, that the endometriosis derives from, and is in phase with, the endometrium [22].

COX-2 overexpression has been described in ectopic endometriosis implants when compared with eutopic endometrium [23], in endometrial carcinoma where it is associated with several parameters of tumor aggressiveness [24,25], and in endometriosis-associated ovarian carcinoma [26].

Moreover, COX-2 has been suggested to be involved in multiple steps in endometrial cancer: besides tumor progression, the enzyme has been implicated in the earlier phases of neoplastic transformation, in endometrial hyperplasia [27] and in endometrial carcinomas arising in endometrial polyps [28].

Accordingly, we found an enhanced positivity for COX-2 both in the tumor and in the neighbouring adenomyotic foci, when compared to the distant adenomyosis and the endometrium.

P53 has been showed to be expressed in endometrial carcinoma with and without adenomyosis [29], in other cases of adenocarcinoma arising from uterine adenomyosis without endometrial malignancy [6,9], but also in hyperplastic and atypical epithelia of carcinoma-associated adenomyosis [29] and in endometrial carcinoma without myometrial invasion [30]. Therefore, an early involvement of p53 in endometrial carcinoma and in the malignant transformation of adenomyosis has been suggested [29, 30]. In the case we present here, p53 positivity was observed in the benign adenomyotic glands, in the atypical hyperplasia and in the neoplasm itself, lending support to the proposed model of carcinogenesis in adenomyosis.

Our case is quite peculiar for the early metastatic spread to the iliac lymph nodes, confirmed by cytological fine-needle aspiration performed during the second-look surgical inspection and by an extensive clinical work-up to exclude other sites of primaries. The origin of the metastasis from adenocarcinoma in adenomyosis was also confirmed by its immunocytochemical profile. A possible explanation for such early metastatic spreading is that this tumor arose in the outer myometrium, near to the richly vascularized parametrium.

In conclusion, considering that no evidence of systemic exogenous or endogenous hyperestrogenism are on record in our case, it is arguable that the presence of a leiomyoma, close to a pool of adenomyotic foci, along with the overexpression of estrogen receptors and enzymes involved in steroidogenesis, represent a hyperestrogenic microenvironment with converging sources of estrogen biosynthesis. Therefore, the same local estrogen excess, besides sustaining adenomyosis and leiomyoma persistence, may also promote growth in tumors having adequate ER/PR expression. This local hormonal overproduction most likely interacts with other events, such as p53 mutations and COX-2 increased activity, in the pathogenesis of adenocarcinoma in adenomyosis.

## Abbreviations

ER: estrogen receptors; PR: progesterone receptors.

## Competing interests

The author(s) declare that they have no competing interests.

## Authors' contributions

**GP **conceived and coordinated the study and drafted the manuscript. **MS **was responsible for the immunohistochemical staining with aromatase and revised the manuscript for important intellectual content. **TP **was responsible for the sampling of the surgical specimen. **KN **was responsible for the immunohistochemical staining with aromatase. **AG **was responsible for the immunohistochemical staining with ER, PR, C-erb B2 and COX-2. **EC **was responsible for performeance of all the surgical interventions. **VC **participated in the coordination of the study and revised the manuscript for important intellectual content. All the authors approved the final manuscript.

## Pre-publication history

The pre-publication history for this paper can be accessed here:


